# Supplementary figures and images for: Predicting polycystic ovary syndrome with machine learning algorithms from electronic health records
Source: Front Endocrinol (Lausanne). 2024 Jan 30;15:1298628. doi: 10.3389/fendo.2024.1298628 (PMC10866556; doi:10.3389/fendo.2024.1298628)

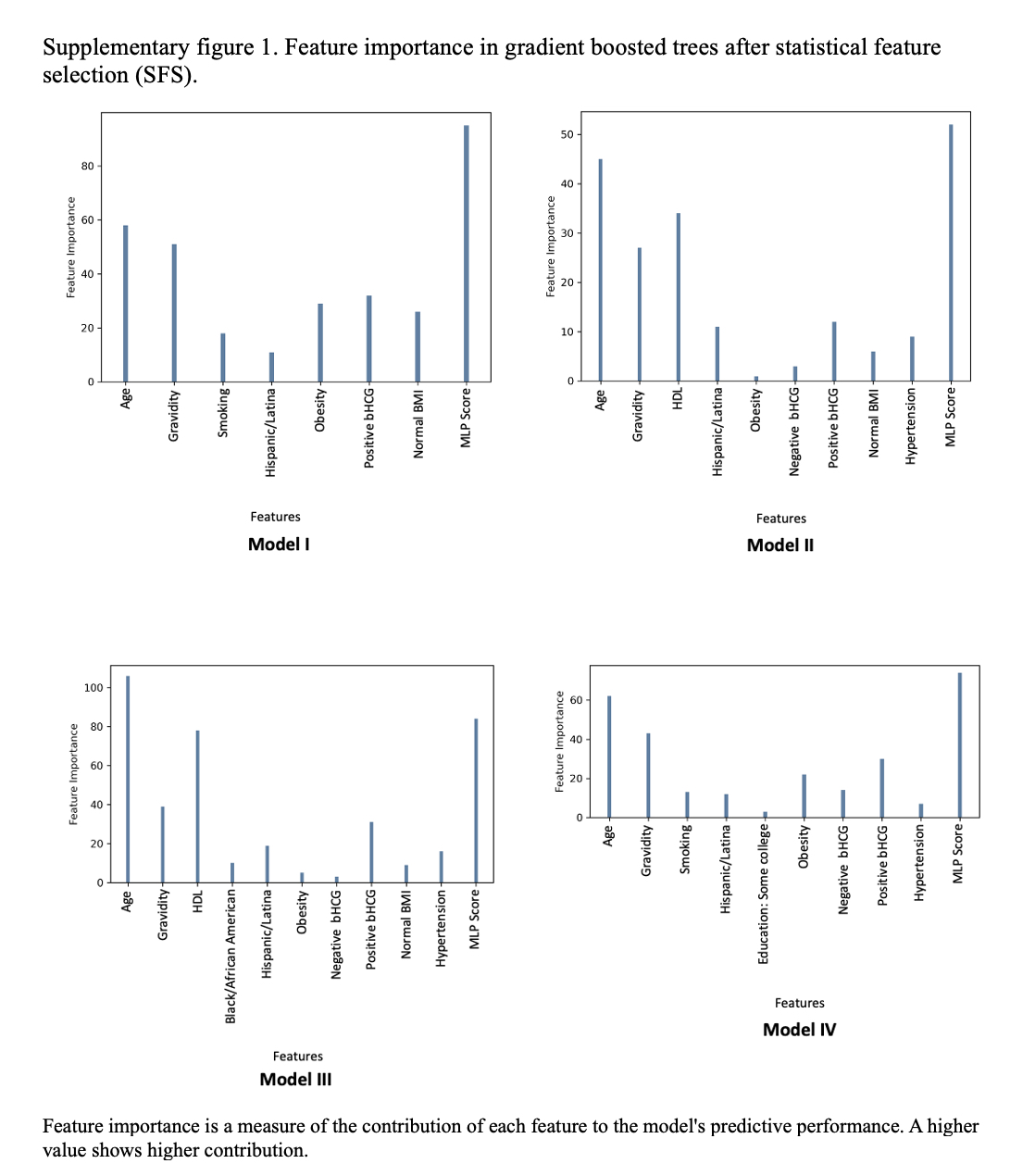

Supplement: Supplementary file 2 [file Image_1.jpeg]
